# Effect of wearing surgical mask during controlled aerobic training on functional capacity and perceived stress in inactive men: a randomized trial

**DOI:** 10.1038/s41598-023-50178-1

**Published:** 2023-12-21

**Authors:** Rana Hesham Mohamed Elbanna, Mariam Omran Grase, Sherif Osama Abdelsalam Elabd, Hend Abd El-Monaem Abd El-Monaem

**Affiliations:** 1 Department of Cardiovascular/Respiratory Disorders and Geriatrics, Faculty of Physical Therapy, Cairo University, Giza, Egypt; 2 Department of Basic Science, Faculty of Physical Therapy Cairo University, Giza, Egypt; 3 Department of Physical Therapy for Internal Medicine and Geriatrics, Faculty of Physical Therapy, May University, Cairo, Egypt

**Keywords:** Health care, Medical research

## Abstract

The study aims to assess the impact of wearing a surgical mask during training on inactive men’s functional capacity and perceived stress. Seventy non-smoker males with body mass index of 25–30 kg/m^2^ and moderate-intensity activity of fewer than 150 min/week were allocated randomly into two equal groups. The surgical mask group performed a controlled endurance exercise while wearing a surgical mask of three protection layers. The mask-less group performed a controlled endurance exercise without wearing any mask. Functional capacity and Perceived Stress were evaluated before and after the intervention. A significant improvement was observed within groups post-intervention in favor of the mask group regarding the Time Up and Go test (*P* < 0.05), with a 15.1% percentage improvement. Post-intervention, there was a significant change in the perceived stress score for the mask and mask-less groups (*P* < 0.05). The improvement in PSS was in favor of mask-less group participants as they changed from being categorized as moderate to mild stress on the PSS, with a 27.1% percentage improvement. Exercising while wearing a surgical mask Positive impacts functional capacity and negatively impacts Perceived Stress in inactive adults. An additional study evaluating the physiological effects of masks on continuous exercise is necessary.

## Introduction

The world is suffering from an unusual life-threatening disaster due to the COVID-19 pandemic. Social distancing and masking have now become a part of daily routine. It is difficult to anticipate when the COVID-19 pandemic will subside and when societies will return to their everyday lives again. It may take some time before that happens. On the other hand, at this time, we do not know the lasting effects of the COVID-19 pandemic on performance as soon as regular life begins to return^1^. Sedentary lifestyles are increasing globally owing to a lack of exercise places, increased job-related sedentary actions such as office work, and prolonged contact with television and video devices^2,3^. Physical inactivity has been detected due to COVID-19 home confinement^4^. Consequences of inactivity include a wide range of chronic diseases comprising a higher mortality level and reduction in overall general health. Throughout history, being sedentary has been unhealthy. Inadequate physical exercise is the fourth most common cause of death. Annually, an estimated 3.2 million mortalities and 32.1% of global disability-adjusted life years (DALYs) are attributed to inadequate physical exercise. Physically inactive people have a 20% to 30% mortality rate higher than those who participate in at least 30 min of moderate-intensity physical exercise on most days of the week^5^. Currently, nearly one-third of the world's population is sedentary, indicating a chief public health problem, so maintaining an active regime during pandemics is essential^6,7^. The COVID-19 outburst has profoundly impacted every aspect of life. Global masking has been proposed as a method of infection control. Regular exercise in a safe environment is an essential strategy for healthy subjects throughout this crisis. Masking may become a necessary part of physical activity as outdoor sports clubs, and open areas may lead to viral transmission^8^. The World Health Organization (2020) emphasizes that individuals should always maintain a physical distance while exercising or walking in a park or other open areas of public. While there is still debate over the safe distance in science, it is vital to take caution when interpreting these early and unpublished data. It is crucial to consider influencing variables in future research, such as the practitioners' head positions while exercising and the direction and speed of the wind^9^. Universal masking should stay up until COVID-19 immunity gets a hold of the disease and vaccination. Subsequently, universal masking should occur in indoor sites where social distancing cannot be preserved, particularly during winter. Mask-wearing will be essential for those people at maximum risk of COVID-19 infection^10^. There is a public debate on whether it is safe for people to exercise while wearing a face mask, even though face masks may allow people to participate in exercise and physical activity throughout the pandemic. For instance, it has been hypothesized that the air trapped in the face mask may impair oxygen intake and increase carbon dioxide rebreathing, causing an increase in the arterial carbon dioxide that displaces the oxygen from hemoglobin^11^. Although some research found that wearing a face mask would have an adverse influence on performance and physiological indicators^12,13^, other studies produced opposite outcomes^14,15^. These divergent study results may be attributable to the significant methodological variations in the relevant literature. Given the absence of evidence-based recommendations for exercising while wearing a face mask, the current study aimed to explore the influence of face masks on functional capacity and perceived stress during controlled aerobic exercise. It was hypothesized that wearing a surgical mask would impair functional capability and perceived stress, particularly in inactive subjects.

## Results

Typically, 70 adult males were allocated randomly into two equal groups. There was no significant variation in age, weight, height, or body mass index (BMI) between the two groups (*P* < 0.05) (Table 1).

**Table 1:** Demographic data and physical characteristics of patients in both groups.

Items	Mask less		Mask		Comparison	
	Mean	± SD	Mean	± SD	t-value	*P* value
Age (years)	21.7	± 2.4	22.5	± 1.8	1.49	0.141
Weight (Kg)	76.7	± 2.9	76.6	± 2.1	0.092	0.927
Height (m)	1.68	± 0.06	1.69	± 0.05	0.520	0.605
BMI (Kg/m^2^)	27.2	± 1.5	26.7	± 1.5	0.656	0.514

*SD* standard deviation, *P* probability, *S* significance, *NS* non-significant.

Regarding the TUG test, utilizing the unpaired T-test, there were no significant differences in pre-intervention between both groups, as the *P* value was 0.981. There was a significant improvement within groups post-intervention in favor of the mask group. Regarding PSS, using Wilcoxon Signed Ranks Test, there was no significant difference pre-intervention between both groups as the *P* value was 0.664. Both groups were suffering from moderate stress on the perceived stress score scale. Employing Independent Samples Mann–Whitney U, there was a significant change in the PSS score for the mask and mask-less groups post-intervention as the *P* value was < 0.05. The improvement in PSS score was in favor of mask-less group participants as the participants in mask-less group intervention changed from being categorized moderate stress to mild stress on PSS (Table 2).

**Table 2:** Comparison between both groups in pre and post-treatment mean Time up and go test and perceived scale score.

	Mask less group		Mask group		*P* value between groups	
	Pre-treatment X ± SD	Post-treatment X ± SD	Pre-treatment X ± SD	Post-treatment X ± SD	Pre-treatment	Post-treatment
TUAGT (s)	9.3 ± 0.8	8.1 ± 0.6	9.3 ± 0.8	7.9 ± 0.7	0.981	0.213
*P* value within groups	*P < 0.05		*P < 0.05			
(% of change)	12.9%		15.1%			
Stress score	16.6 ± 1.4	12.1 ± 0.9	16.8 ± 1.4	14.9 ± 0.8	0.616	*P < 0.05
Score range	(14–19)	(10–13)	(14–19)	(14–16)		
*P* value within groups	*P < 0.05		*P < 0.05			
(% of change)	27.1%		11.3%			

*X* mean, *SD* standard deviation.

*P < 0.05 is statistically significant.

## Discussion

Although facemasks are frequently used by many individuals, such as healthcare professionals and employees in many industrial settings, as an effective tool to guard against infections and viral transmission, it is unknown if prolonged usage of these facemasks might have adverse consequences. The effect of wearing a surgical mask on functional capacity and perceived stress, particularly in response to indoor aerobic exercise, in sedentary males, has not been studied to our knowledge. Putting on a face mask in public areas hinders the spread of infectious diseases by avoiding the breath of infectious droplets. While the consequence of decreasing the hazard of the spread of respiratory viruses remains debated, wearing face masks is considered the main way to avoid the spread of respiratory viruses among people in everyday life^16^. Exercising in any public place should be encouraged nowadays. On the other hand, universal recommendations strongly boost the display of nose and mouth facial coverings to aid in limiting the transmission of COVID-19^17^. Wearing masks is linked to a significant modest fall in spirometry and cardiorespiratory variables at rest and during training. This effect is owing to increased airflow resistance. However, as exercise ventilator restriction is far from being achieved, using face masks during a maximal workout is safe^18^. Even though the hemodynamic and hematologic functions of the two mask groups (N95 and surgical) were comparable after exercise, these results show that wearing a mask, especially a surgical mask, while exercising during the ongoing pandemic is safe and doesn't put a person's health at risk^12^. In healthy individuals, performing moderate to strenuous aerobic bodily activity while wearing a mask is practical and safe and results in only minor variations in physiological parameters^8^. Wearing an Elevation Training Mask while performing maximal incremental cycling tests leads to a marked increase in brain oxygenation without any alteration in skeletal muscle oxygenation^19^.

On the other hand, a recent study included 12 healthcare professionals who underwent a cardiopulmonary exercise test while wearing a surgical mask, an N95 mask, or no mask at all. For both surgical and N95 masks, the degree of dyspnea worsened and exercise time was reduced. In contrast to wearing no mask, Cardiopulmonary function is somewhat adversely affected by surgical mask use, and this effect is more severe when using an N95 mask^20^. Once again, the impacts of wearing no mask, a surgical mask, and an N95 mask on 12 healthy men were measured in a prospective cross-over study. Surgical masks decrease comfort, the ability for cardiopulmonary activity, and ventilation in healthy individuals. Medical face masks significantly reduce cardiopulmonary capacity, which considerably reduces the ability to perform demanding physical and vocational tasks^21^. Fabric facial masks cause a 14% decrease in exercise time and a 29% reduction in VO2 max, which is attributed to the perceived distress related to mask use. Individuals who wore a fabric face mask reported feeling increasingly short of breath at increased workout intensities when compared to those who did not wear a mask^22^. Using surgical masks during training elevates the blood lactate concentration, rate of perceived exertion, and perceived stress in 50 and 400 m maximal running tests^23^. The current research has some limitations that must be noted. First, since only sedentary, nonsmoking males were involved in this study, the results cannot be generalized to clinical populations. This is relevant because, in certain clinical groups, tiny but considerable increases in cardiac effort may pose greater cardiovascular stress and may disguise the possible negative impact of wearing a face mask while exercising. Second, in the current investigation, only the acute impact of wearing a mask during exercise was investigated; it remains to be determined if regular exercise while wearing a mask will result in altered physiological and functional responses to exercise. The current study only looked at people between 18 and 25 years old. It needs to be found out if similar results can be found in younger and older groups.

## Material and methods

### Study design

A parallel randomized controlled endurance exercise intervention trial on sedentary men in the Outpatient Clinic of Cairo University's Faculty of Physical Therapy. Written informed consent was obtained from every enrolled patient or their guardians

### Inclusion criteria

Inactive non-smoking men (less than 150 min of moderate-intensity activity per week)^5^ aged between 18 and 25 years with a BMI of 25–30 kg/cm^2^ are capable of doing a bodily activity as assessed by physician screening.

### Exclusion criteria

Participants were excluded if they met one or more of the following criteria: uncontrolled diabetes mellitus, uncontrolled hypertension, hemoglobinopathies, class II, III, morbid obesity, mentally unstable people, arterial oxygenation (SpO2 < 95%) using pulse oximetry, any orthopedic disorders, and any neurologic disorders.

### Randomization

The allocation was indicated by a code printed on pre-made cards used for randomization. After completing all baseline assessments, these cards were placed in opaque, sealed envelopes. A simple randomization sequence was conducted by an investigator who was not directly involved with the recruitment, assessment, or management of the patients.

### Participants

**Figure 1:**
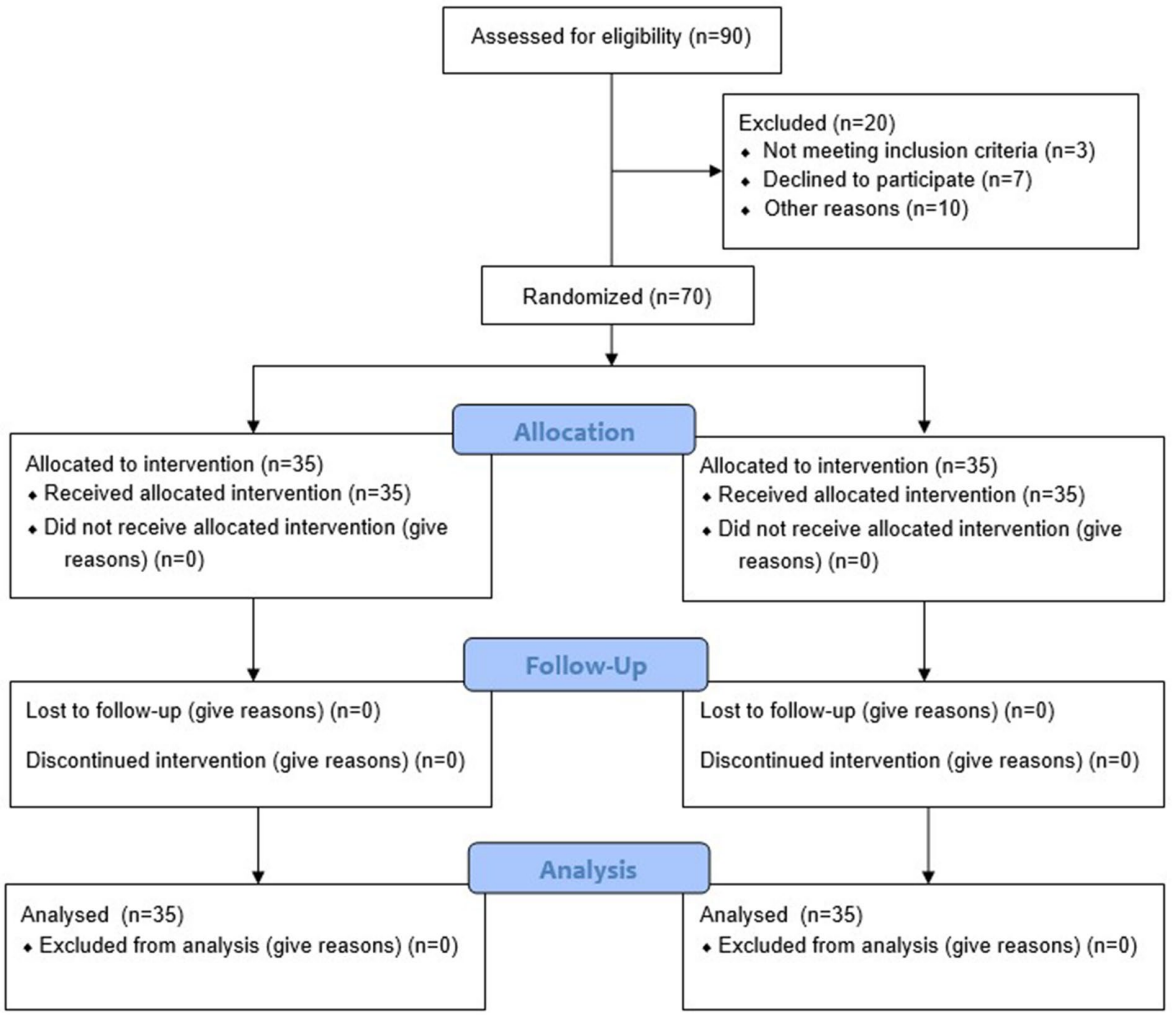
Study flow chart.

Ninety volunteers were recruited through the Outpatient Clinic of Cairo University's Faculty of Physical Therapy. The sample size was calculated using the G.Power program version 3.1. From these, 70 were selected to participate according to the inclusion criteria. However, during the study, ten participants were excluded for the following reasons: seven participants refused to participate in the first session, two participants had knee pain, one participant experienced chest heaviness in the first session, and ten were excluded at the study's outset for different reasons. The study consort flow chart is represented in Fig. 1. Thus, the final sample size was 70 volunteers divided into the following groups:

The surgical mask group performed a controlled endurance exercise while wearing a suitable fit surgical mask with three layers of protection (n = 35). Controlled exercise performed within the symptom or exertion threshold of participants. It is based on physically experiences such as increased heart rate, increased respiration or breathing rate, increased perspiration, and muscular exhaustion that occur during physical exhaustion.

The mask-less group will perform a controlled endurance exercise without wearing masks (n = 35).

### Interventions

Participants were asked to work out 5 days per week for 15–30 min for 4 weeks, in addition to 5 min of warm-up and 5 min of cool-down. Three days/week, the training intervention was under the supervision of a physiotherapist (gradually increased from 50% heart rate reserve to 80% heart rate reserve following the Karvonen method)^24^. The other 2 days/week of exercise was unsupervised and consisted of brisk walking at a location determined by the participant and approved by the physiotherapist for 15–30 min. Weekly activity logs were used to track exercise adherence. Strap heart rate monitors were used to ensure that at least half of the exercise sessions were completed within the target heart rate zone. Endurance training was performed on the Horizon Fitness T101 Treadmill.

### Primary outcome measures

#### Functional capacity

TUG test is a functional mobility test that allows healthcare practitioners to evaluate a person's mobility on a daily basis^25^. During the TUG, the person stands up from a chair, walks 3 m to a marker, turns around, walks back to the chair, and sits down. This test, therefore, consists of two 180° turns and 6 m of walking^23^. Participants were asked to wear their usual footwear. The starting point of the test began when the participant sat back in a typical armchair and recognized a three-meter line on the floor. The participants were asked to stand up, walk to the line on the ground at their normal stride, turn, walk back to the chair at their normal pace, and sit down another time.

#### Perceived stress

Perceived stress was measured utilizing the Perceived Stress Scale (PSS)^26^. PSS is a definitive stress assessment instrument consisting of ten questions. It remains a popular choice to understand how dissimilar situations affect our emotional state and perceived stress. Participant scores on the PSS can range from 0 to 40, with higher scores demonstrating greater perceived stress. A single physical therapy senior took a comprehensive history and performed a clinical assessment for all patients. A baseline assessment was done pre-intervention and 4 weeks’ post-intervention and on a separate day from assessing functional capacity using the TUG test.

### Statistical analysis

This study statistical analysis was conducted employing SPSS version 22. Descriptive statistics for the mean and standard deviation of age, height, and BMI were calculated employing an unpaired T-test. Significant changes in Time up and go test before and after the intervention within groups were analyzed utilizing Paired Sample T-test, and the differences between groups were detected using MANOVA. Significance changes in PSS scores within groups were analyzed using Wilcoxon Signed Ranks Test, but the differences between groups before and after intervention were analyzed utilizing the Independent Samples Mann–Whitney U test. (α = 0.05) was the level of significance.

### Ethics approval and consent to participate

The study protocol was authorized by the Ethical Committee through the Faculty of physical therapy at Cairo University (No: P.T.REC/012/003277); and registered on the clinicaltrials.gov website with identification number: (NCT05004480) at the date of 13/08/2021. The trial was performed between the middle of June 2021 and the beginning of December 2021. The study ended after it was completed. All methods were performed in accordance with relevant guidelines and regulations. Written informed consent was obtained from every enrolled patient or their guardians.

## Conclusion

In conclusion, using face masks during controlled endurance exercise has an influence on functional ability and daily mobility. Maintaining a regular exercise program may reduce the risk of disorders such as obesity, diabetes, and cardiovascular disease in a long-term pandemic. Wearing a surgical mask during supervised exercise can increase functional capacity and mobility, but it has a negative impact on perceived stress, according to our findings. An additional study evaluating the physiological effects of masks on continuous exercise is necessary.

## Data Availability

The databases used and analyzed during the current study are available from the corresponding author on reasonable request.
